# Deep Conservation of Histone Variants in Thermococcales Archaea

**DOI:** 10.1093/gbe/evab274

**Published:** 2021-12-11

**Authors:** Kathryn M Stevens, Antoine Hocher, Tobias Warnecke

**Affiliations:** Epigenetics Section, Medical Research Council London Institute of Medical Sciences, United Kingdom; Institute of Clinical Sciences, Faculty of Medicine, Imperial College London, United Kingdom; Epigenetics Section, Medical Research Council London Institute of Medical Sciences, United Kingdom; Institute of Clinical Sciences, Faculty of Medicine, Imperial College London, United Kingdom; Epigenetics Section, Medical Research Council London Institute of Medical Sciences, United Kingdom; Institute of Clinical Sciences, Faculty of Medicine, Imperial College London, United Kingdom

**Keywords:** archaea, chromatin, histones, paralogs, Thermococcus

## Abstract

Histones are ubiquitous in eukaryotes where they assemble into nucleosomes, binding and wrapping DNA to form chromatin. One process to modify chromatin and regulate DNA accessibility is the replacement of histones in the nucleosome with paralogous variants. Histones are also present in archaea but whether and how histone variants contribute to the generation of different physiologically relevant chromatin states in these organisms remains largely unknown. Conservation of paralogs with distinct properties can provide *prima facie* evidence for defined functional roles. We recently revealed deep conservation of histone paralogs with different properties in the Methanobacteriales, but little is known experimentally about these histones. In contrast, the two histones of the model archaeon *Thermococcus kodakarensis*, HTkA and HTkB, have been examined in some depth, both in vitro and in vivo. HTkA and HTkB exhibit distinct DNA-binding behaviors and elicit unique transcriptional responses when deleted. Here, we consider the evolution of HTkA/B and their orthologs across the order Thermococcales. We find histones with signature HTkA- and HTkB-like properties to be present in almost all Thermococcales genomes. Phylogenetic analysis indicates the presence of one HTkA- and one HTkB-like histone in the ancestor of Thermococcales and long-term maintenance of these two paralogs throughout Thermococcales diversification. Our results support the notion that archaea and eukaryotes have convergently evolved histone variants that carry out distinct adaptive functions. Intriguingly, we also detect more highly diverged histone-fold proteins, related to those found in some bacteria, in several Thermococcales genomes. The functions of these bacteria-type histones remain unknown, but structural modeling suggests that they can form heterodimers with HTkA/B-like histones.

Significance Histones are key components of chromatin in eukaryotes and many archaea. In eukaryotes, histone variants exist, and play defined roles in cellular function and development. Some of these variants are highly conserved and can date back to the last common ancestor of eukaryotes. Archaea also frequently encode multiple sequence-divergent histones but whether these play distinct functional roles that are conserved through evolution remains largely unknown. Here, using phylogenetic tools, we establish the existence of histone variants with different properties in the order Thermococcales, which have been conserved for hundreds of millions of years. Our work provides additional evidence for histone variants in archaea and that these have evolved in parallel to eukaryotes to mediate flexible, adaptive chromatin states.

## Introduction

The ability of eukaryotic cells to respond to environmental change and regulate transcription relies on dynamic control of DNA accessibility through chromatin alterations. This involves many different processes, including the addition/removal of histone modifications and the exchange of histone proteins for paralogous variants. Such variants can modify structural properties of the nucleosome or change how it interacts with its binding partners (Talbert and Henikoff 2010; Henikoff and Smith 2015; Martire and Banaszynski 2020). For example, macro-H2A has a large C-terminal domain and precipitates transcriptional repression (Bönisch and Hake 2012; Martire and Banaszynski 2020), whereas cenH3, a fast-evolving H3 variant, is specifically localized to centromeres and involved in chromosome segregation (Palmer et al. 1991; Talbert and Henikoff 2010). Importantly, significant functional changes can come from small differences in sequence. H3.3, for example, is deposited in a replication-independent manner in actively transcribed regions of the genome (Talbert and Henikoff 2010) and is important for mammalian development (Sitbon et al. 2020; Jang et al. 2015) but differs from its paralog H3.1 by only five amino acids.

Histones are not exclusive to eukaryotes. Archaeal histone proteins, first discovered in *Methanothermus fervidus* (Sandman et al. 1990; Starich et al. 1996), have since been identified in diverse archaeal lineages (Henneman et al. 2018; Hocher et al. 2021) and are often highly expressed (Hocher et al. 2021). Eukaryotic and archaeal histones share a conserved histone fold (HF) domain, form dimers and tetramers that are structurally very similar, and bind DNA nonspecifically, albeit with a preference for more bendable sequences (Bailey et al. 2000; Decanniere et al. 2000; Nalabothula et al. 2013; Mattiroli et al. 2017; Rojec et al. 2019). Unlike eukaryotic histones, almost all archaeal histones lack long terminal extensions (“tails”) (Henneman et al. 2018). In at least some instances, archaeal histones have the capacity to form homo- as well as heterodimers and to assemble into long oligomeric structures on DNA (Mattiroli et al. 2017). These extended complexes can, in theory, consist of different histone paralogs, providing opportunities for chromatin state modulation through the exchange of histones with different properties (Stevens et al. 2020). In fact, many archaea encode two or more sequence-divergent histone paralogs, but whether these paralogs have defined functional roles akin to eukaryotic histone variants, and whether their expression and assembly change dynamically to mediate adaptive chromatin states, remains poorly understood.

What we do know from prior experimental work is that archaeal histone paralogs are more than mere copy number variants. The two histones of *M. fervidus* (HMfA, HMfB), for example, display differences in DNA-binding affinity (Bailey et al. 2002). Compared with HMfA, recombinant HMfB also induces more positive supercoiling upon binding to plasmid DNA and forms a more compact complex as inferred from gel-shift and tethered particle motion experiments (Sandman et al. 1994; Henneman et al. 2021). There are also differences between HMfA and HMfB in their relative expression during the growth cycle: in early exponential phase, HMfA is more highly expressed than HMfB, expression of which increases toward stationary phase to reach an almost equal ratio between the two (Sandman et al. 1994). The different properties of *M. fervidus* histones are consistent with the hypothesis that the two paralogs may have distinct functions, but whether the properties are physiologically relevant and affect organismal fitness remains to be addressed experimentally.

Recently, we considered this question using an evolutionary approach. We identified histone paralogs in the order Methanobacteriales that exhibit distinct structural properties and have been maintained over hundreds of millions of years (Stevens et al. 2020), indicative of the importance of each individual paralog for fitness. Structural modeling identified histone variants that prevent stable tetramerization and might act as *capstones* that limit further extension when incorporated into a histone oligomer, providing a potential pathway to dynamically alter chromatin state. Are the Methanobacteriales unique or are there other clades of archaea with histone paralogs that have been maintained over long periods of time? And do these paralogs also show conserved and distinct structural properties?

Here, we consider archaea in the order Thermococcales, which includes the model archaea *Pyrococcus furiosus* and *Pyrococcus abyssi* as well as *T. kodakarensis*, which has served as a model species for the in vivo study of archaeal histones. Thanks to the efforts of Santangelo and coworkers in particular, its two histones—HTkA (TK1413) and HTkB (TK2289)—are arguably the best characterized paralogs in vivo. Similar to HMfA and HMfB in *M. fervidus*, HTkA and HTkB can assemble into long oligomers both in vitro and in vivo (Maruyama et al. 2013; Mattiroli et al. 2017; Sanders et al. 2021; Bowerman et al. 2021). The two histones differ from one another at 11 out of 67 residues (84% identity) and have several distinct properties. HTkA is the more highly expressed paralog, at least in exponential phase, where it makes up 1.1% of the proteome compared with 0.66% for HTkB (Hocher et al. 2021). Together, they are abundant enough to coat the entire *T. kodakarensis* genome (Sanders et al. 2019). HTkB has been shown to bind to DNA more strongly than HTkA and to form more compact complexes, which show faster migration during agarose gel electrophoresis (Higashibata et al. 1999). Deletion of each histone individually results in overlapping but distinct perturbations of the transcriptome (Čuboňová et al. 2012; Sanders et al. 2021). Notably, HTkB-deficient cells exhibit reduced growth, possibly due to changes in the expression, not seen in strains lacking HTkA, of genes that encode translation factors and ribosomal proteins (Čuboňová et al. 2012). Deletion of *htkA* but not *htkB* leads to downregulation of hypothetical membrane proteins and prevents transformation of *T. kodakarensis*, suggesting HTkA alone plays a critical role in DNA uptake and/or integration (Čuboňová et al. 2012).

In this study, we show that histone paralogs with HTkA- and HTkB-like properties are present across the Thermococcales, including Thermococcus, Pyrococcus, and Palaeococcus spp. We use structural modeling to show that, in most Thermococcales, HTkB-like histones are predicted to exhibit stronger DNA binding than those with HTkA-like properties. Phylogenetic analysis reveals that HTkA-like histones share a common ancestor to the exclusion of HTkB-like histones and vice versa, suggesting that the last common ancestor of the Thermococcales already encoded an HTkA-like and an HTkB-like histone, each of which has been maintained throughout the diversification of this clade for (very) approximately 750 Myr (Wolfe and Fournier 2018). The long-term preservation of these two paralogs across the order Thermococcales supports the notion that HTkA/B in *T. kodakarensis* (and their orthologs in other Thermococcales) make unique contributions to genome function and fitness. These findings add further evidence that histone variants are widespread in archaea, evolving in parallel to those in eukaryotes.

Intriguingly, many Thermococcales archaea encode additional types of histone-fold proteins that are similar to histone-fold proteins found in some bacteria (Alva and Lupas 2019). One of these consists of an end-to-end duplication of the HF and is rarely found in archaea outside the Thermococcales. Their physiological roles remain unknown, but structural modeling suggest that they are able to heterodimerize with HTkA/B and might therefore further diversify histone-based chromatin states in these archaea.

## Results and Discussion

### Almost All Thermococcales Have Histone Paralogs with HTkA- and HTkB-Like Properties

**Fig. 1.**
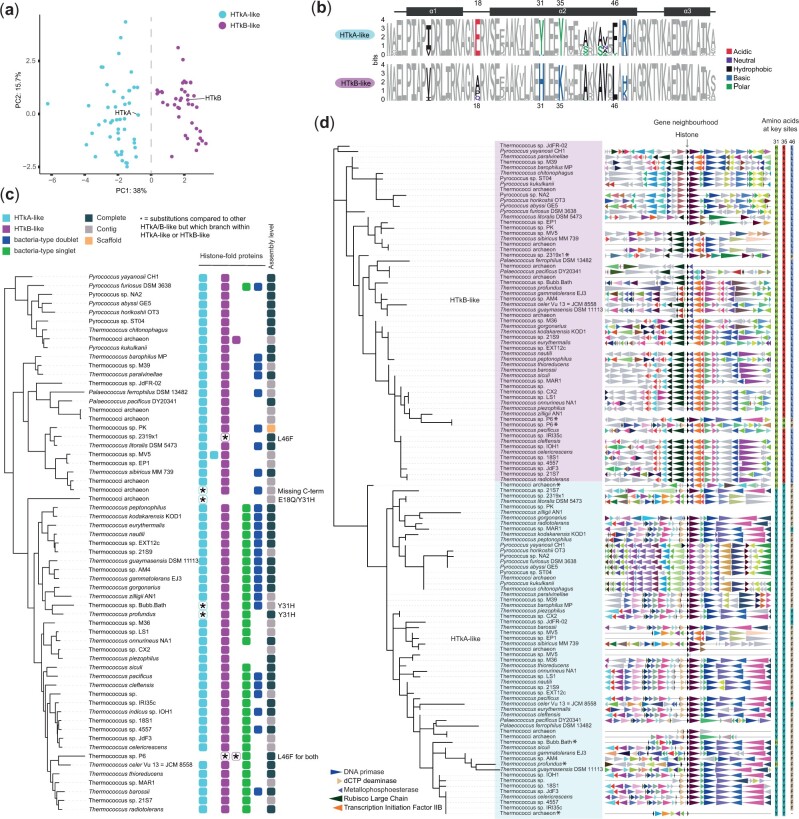
(*a*) Principal component analysis of Thermococcales HMf-like histones based on amino acid properties (AAStats, see Materials and Methods). Histones that cluster with either HTkA or HTkB along the first principal component are colored accordingly (HTkA-like histones in blue, HTkB-like in purple). (*b*) Sequence logos showing amino acid composition of HTkA- and HTkB-like histones across 61 Thermococcales. Amino acids that differ substantially between the two groups are colored. Positions are numbered relative to HMfB from *Methanothermus fervidus* to facilitate comparison with prior studies. (*c*) GTDB species tree for all Thermococcales in the data set indicating presence/absence of histones of a particular type in each genome. Each square represents one histone and is colored by histone type. (*d*) Protein-level phylogenetic tree of all HMf-like Thermococcales histones. Genes in the neighborhood are colored to indicate ortholog identity (see Materials and Methods). Note that, while the gene neighborhood is broadly conserved for HTkA- vis-à-vis HTkB-like orthologs, some HTkB-like genes (e.g., in *Thermococcus chitonophagus*) have a 3′ neighborhood normally found for HTkA. This is owing to a large genomic rearrangement event in which HTkB served as the breakpoint (not shown).

To identify putative histone proteins across the Thermococcales, we scanned 61 predicted proteomes using HMM models and iterative jackhmmer searches (see Materials and Methods). Histones with a single histone-fold domain similar to archaeal HMf-like histones were found in all genomes (fig. 1*c*, supplementary table S1, Supplementary Material online). We also recovered putative histone-fold proteins similar to those found in some bacteria (Alva and Lupas 2019), which we discuss further below. A principal component analysis of the HMf-like archaeal histones based on their amino acid properties and isoelectric points (see Materials and Methods) suggests that histones can be assigned to one of two groups. One of these groups contains HTkA, the other HTkB (fig. 1*a*). This is consistent with a previous classification effort that also recovered two major groups of Thermococcales histones (Henneman 2019). Amino acid identities at several residues along the HF differ systematically between groups and are diagnostic of group membership. For example, tyrosine is always found at position 35 (Y35) in HTkA-like histones whereas HTkB-like histones have a positively charged lysine (K, 60 out of 62) or histidine (H, 2 out of 62). Similarly, glutamic acid at position 18 (E18) is present in 59 out of the 61 HTkA-like but none of the HTkB-like histones (fig. 1*b*).

For some of these residues, we know from prior in vitro studies—as well as from structural modeling—that amino acid identity can affect specific histone properties (Soares et al. 2000; Stevens et al. 2020). For example, substituting Y for K at residue 35 (the amino acids seen in HTkA- and HTkB-like histones, respectively), increases the stability of recombinant histone HFoB from *Methanobacterium formicicum* (Li et al. 2000). In addition, evidence from mass spectrometry indicates that K35 in HTkB from *T. kodakarensis* and *Thermococcus gammatolerans* is acetylated in vivo (Alpha-Bazin et al. 2021) although stoichiometry and functional significance of this modification remain to be determined. A tyrosine at the same position in HTkA removes the potential for acetylation. E18 in HMfB forms an intermolecular salt bridge with K53, which helps to stabilize the interaction between monomers in the histone dimer (Decanniere et al. 2000; Sandman et al. 2001). Mutating E18 to proline does not alter DNA binding (Soares et al. 2000), but loss of the intermonomer salt bridge may result in less rigid dimer structures. Finally, having leucine (L) or phenylalanine (F) at residue 46 has no obvious effect on DNA binding in HMfA/B, but the residue, located at the interface between dimers, is important for tetramer formation (Soares et al. 2000; Marc et al. 2002).

**Fig. 2.**
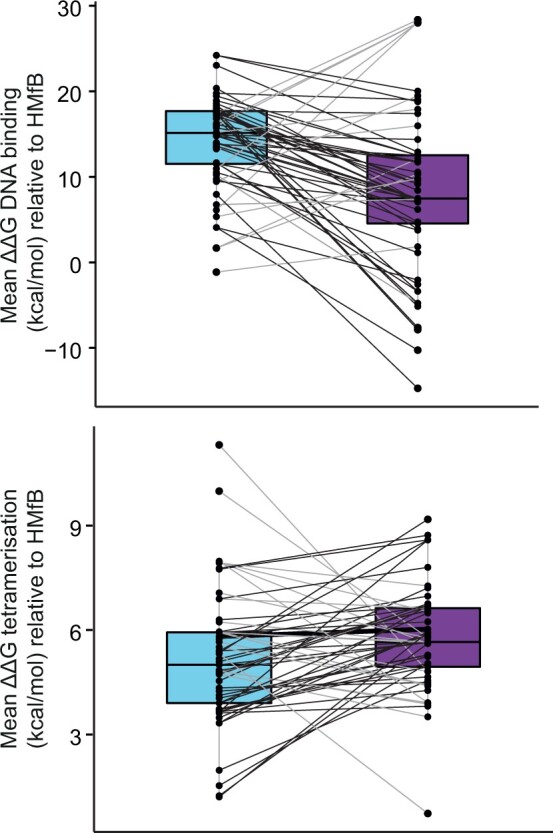
Predicted DNA-binding affinity (top) and tetramer stability (bottom) for HTkA/B-like paralogs. Lines connect paralogs from the same genome. Lines are black when DNA-binding affinity is stronger or tetramer interface energy weaker, respectively, for the HTkB-like paralog.

We considered how amino acid differences between HTkA- and HTkB-like histones affect two key aspects of the histone–DNA complex: DNA affinity and tetramerization strength, a proxy for tetramer stability. Using a structural modeling approach, we find that predicted DNA binding for HTkB-like paralogs is, in most cases, stronger than for HTkA-like paralogs (fig. 2, see Materials and Methods). This is in line with experimental findings that HTkB binds to DNA more tightly and forms a more compact complex with DNA than HTkA (Higashibata et al. 1999). In contrast, predicted tetramerization strength does not strongly discriminate HTkA- from HTkB-like histones (fig. 2). We also considered residues (K14, G17, K26, E30, E34, Q48, E58, K61, K65) that were previously suggested to be important for stacking interactions for either HTkA or HTkB, in the context of longer oligomers (Mattiroli et al. 2017; Henneman et al. 2018, 2021). None of these residues differ substantially between HTkA- and HTkB-like histones, with the exception of Q48 (HTkB) (Henneman et al. 2018). We note that Q48 is relatively uncommon in HTkB-like histones and that *T. kodakarensis* HTkB may therefore form more stable oligomeric complexes than the HTkB-like histones of most of its cousins.

### HTkA- and HTkB-Like Histones Form Ancient Paralogous Groups

Almost all Thermococcales have both an HTkA-like and an HTkB-like histone (fig. 1*c*). This is consistent with (but not sufficient to demonstrate) ancient paralogy. To unravel the evolutionary history of Thermoccoccales histones, we used RaxML-NG (Kozlov et al. 2019) to build phylogenetic trees of all 123 HMf-like histones found across the 61 genomes in our analysis (see Materials and Methods). We find that HTkA-like and HTkB-like paralogs neatly separate into two groups defined by their position on the tree (fig. 1*d*). This pattern of separation indicates that one HTkA- and one HTkB-like histone were present in the last common ancestor of Thermococcales. We detect only a small number of lineage-specific duplications and deletions and find no evidence of rampant horizontal gene transfer (fig. 1*c*). The observation that both paralogs have been maintained along divergent Thermococcales lineages strongly suggests that at least some of the amino acid differences between them are functionally important and under selection. Along with our recent report of ancient histone paralogs in the Methanobacteriales (Stevens et al. 2020), this finding provides further evidence that histone variants exist in archaea, evolving in parallel to those in eukaryotes. Note that, at present, we have no convincing evidence that histone paralogs in the Thermococcales and those found in the Methanobacteriales arose from the same ancient duplication event.

### Some Thermococcales Encode Histone-Fold Proteins Similar to Those Found in Bacteria

**Fig. 3.**
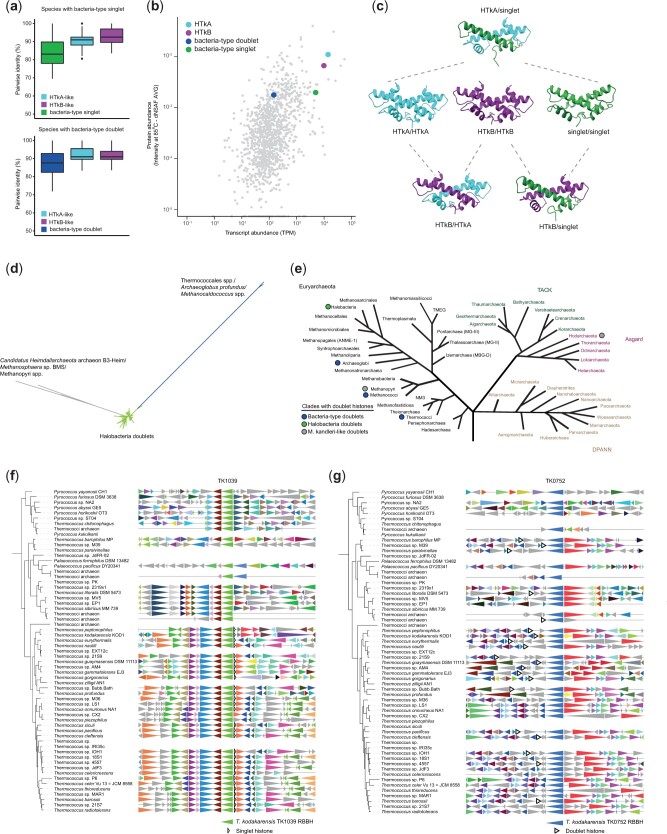
(*a*) Pairwise identity for histones in species with HTkA-like, HTkB-like, and bacteria-type singlet (top) or doublet histones (bottom) across all Thermococcales. (b) Protein and transcript abundance (see Materials and Methods) of genes in *T. kodakarensis.* TPM: transcripts per million. (c) AlphaFold-predicted homodimeric structures of TK1040 (green), HTkA (light blue), and HTkB (purple) and heterodimers of HTkA and HTkB (light blue/purple), HTkA and the bacteria-type singlet TK1040 (light blue/green), and HTkB and TK1040 (purple/green). (*d*) Protein-level tree for doublet histones (containing an end-to-end duplication) in archaea. Green branches contain proteins from Halobacteria, blue branches contain bacteria-type doublet histones in Thermococcales, *Methanocaldococcus* spp. and one *Archaeglobus* sp., gray branches show doublet histones including HMk from *Methanopyrus kandleri* (Slesarev et al. 1998; Fahrner et al. 2001). (*e*) Clades with doublet histones highlighted on an tree capturing archaeal diversity, adapted from Borrel et al. (2020) and Stevens et al. (2020) to include the clade for the species *Candidatus Heimdallarchaeota* archaeon B3-Heim, more recently suggested to be a member of the Hodarchaeota (Liu et al. 2021). Circles denote clades containing a doublet histone and are colored by the type of doublet (see *d*). (*f, g*) Thermococcales GTDB species tree showing syntenic regions for TK1039 (light green, in *f*) and TK0752 (blue, in *g*) commonly found located close to the bacteria-type singlet or doublet histones. Genes are colored to indicate homology (see fig. 1*d* and Materials and Methods).

Our survey also revealed that, alongside the HTkA/B-like histones, many Thermococcales genomes encode histone-fold proteins similar to those found in some bacteria (fig. 1*c*, supplementary table S1, Supplementary Material online), which harbor either a single or two (pseudodimeric) HF domains (Alva and Lupas 2019). We will refer to these as bacteria-type singlets and doublets, respectively. Both types are, on average, less well conserved than HTkA/B-like histones (fig. 3*a*) and their distribution across the Thermococcales is noticeably patchier (figs. 1*c* and 3*f* and *g*). Neither type is present in the closest sister clades (Methanofastidiosa, Theionarchaea).

The bacteria-type singlet is confined to a monophyletic group that comprises some (but not all) *Thermococcus* spp. (figs. 1*c* and 3*f*). It is present in all these species in the same highly conserved syntenic context, suggesting a single origin (fig. 3*f*). Following acquisition, the histone has been maintained along almost all lineages. There is only a single loss event (a clean deletion) in the branch leading to *Thermococcus piezophilus* (fig. 3*f*). Conserved synteny is also consistent with a single, earlier origin for the bacteria-type doublet, with multiple subsequent losses (figs. 1*c* and 3*g*). Based on the branching patterns and different syntenic context, the bacteria-type histones in *Pyrococcus furiosus* have likely been acquired secondarily from an extant or ancient *Thermococcus* species.

Bacteria-type doublets differ considerably in sequence from doublet histones previously described in haloarchaea and *Methanopyrus kandleri* (fig. 3*d*). Outside the Thermococcales, we only detect additional bacteria-type doublets in some (hyper)thermophilic Methanocaldococcus and Archaeoglobus species, but never their mesophilic relatives, suggestive of horizontal gene transfer in a high-temperature niche.

Bacteria-type histone-fold proteins have only recently been recognized and await functional characterization. The only functional data we have at present comes from transcriptome/proteome profiling. Consistent with lower sequence-level conservation (fig. 3*a*), the relative expression levels of these genes in *T. kodakarensis* (singlet: TK1040; doublet: TK0750) are lower than those of HTkA/B-like histones at both the transcript and protein levels (fig. 3*b*; Jäger et al. 2014; Sas-Chen et al. 2020). Together, they make up 0.37% of the measured exponential-phase proteome compared with 0.66% for HTkB and 1.1% for HTkA. TK1040 was previously identified in *T. kodakarensis* chromatin fractions (Maruyama et al. 2011), suggesting a (direct or indirect) association with DNA. However, the same study estimated that <1% of the amount of chromatin-associated proteins were attributable to TK1040. We therefore consider it unlikely that these histones are global organizers of DNA similar to HTkA/B-like histones, but might modulate chromatin state, either locally or globally, in response to environmental change. In both *P. furiosus* and *T. kodakarensis*, the bacterial-type doublets are under the control of the heat shock regulator Phr (encoded by PF1790 and TK2291, respectively) and upregulated upon Phr deletion, suggesting a potential role in response to heat shock in these archaea (Kanai et al. 2010; Keese et al. 2010). The *T. kodakarensis* doublet is also downregulated at lower temperatures, similar to HTkA/B (Hocher et al. 2021), further consistent with a role in temperature adaptation.

Can these HF proteins interact with HTkA/B-like histones? We used AlphaFold (Jumper et al. 2021) to predict the structure of combinations of HTkA, HTkB and the bacterial HF singlet from *T. kodakarensis*. Using this approach, all three are predicted to form homodimers and, as expected, HTkA and HTkB form a stable heterodimer. When a combination of either HTkA or HTkB and the bacterial singlet are used, Alphafold also predicts that these will form a heterodimer (fig. 3*c*). The presence of non-HMf-like HF proteins in Thermococcales genomes adds to the potential functional diversity of histone-based chromatin in these species and may dynamically alter DNA accessibility at different stages of cell growth or in response to environmental challenges. Further experimental investigation is required, however, before meaningful conclusions can be drawn in this regard, including whether they do interact, both structurally and functionally, with the HTkA/B-like histones.

## Materials and Methods

### Identification of Histones in Thermococcales Genomes

Protein sets, genomes, and GFF files for all available genomes of class Thermococci were downloaded from GenBank (https://www.ncbi.nlm.nih.gov/assembly) using taxid 183968 (accessed on May 27, 2021). Genomes not present in the GTDB tree (Parks et al. 2021) (https://gtdb.ecogenomic.org, accessed August 1, 2021, see below) were removed. Two species that were annotated as Thermococci in NCBI but branched outside the main group on the GTDB tree were removed from the analysis, leaving a final set of 61 genomes, all from the order Thermococcales. Protein sequences were predicted using Prodigal v2.6.3 (Hyatt et al. 2010) where not provided through GenBank. Histone proteins were extracted from the protein sets through HMM searches using HMMER v3.3.1 (hmmsearch–noali (Eddy 2011; Finn et al. 2011) using Pfam models CBFD_NFYB_HMF and DUF1931 (Finn et al. 2014) as well as a Jackhmmer searches using the singlet and doublet histones from bacteria as a seed (Alva and Lupas 2019). Bacteria-type histones hit in the initial search were used to build an HMM model and the Thermococcales protein set was re-searched using HMMER v3.3.1 as above (hmmsearch --noali). Some proteins incorrectly identified as histones at this stage were manually filtered out.

### Classification of Thermococcales Histones into HTkA-Like and HTkB-Like Groups

HMf-like histones in Thermococcales downloaded from GenBank (accessed on May 27, 2021) (see above) were aligned using MAFFT (Katoh and Standley 2013) (–localpair --maxiterate 1000). Histones were clustered based on amino acid composition of their peptide sequences using AAStats from the R package Seqinr (Charif and Lobry 2007). Histones that clustered with HTkA and HTkB were assigned HTkA- or HTkB-like status, respectively (see fig. 1*a*). Twenty maximum likelihood phylogenetic trees were built using Raxml-NG (Kozlov et al. 2019) with the LG+G4 model of evolution as suggested by ModelTest-NG (Darriba et al. 2020). The unrooted best maximum likelihood tree is shown. All trees were plotted using iTOL (Letunic and Bork 2019). Orthologous genes in the genomic neighborhood of each histone were highlighted on the tree using Genespy as best reciprocal hits (Garcia et al. 2019). Orthologs in the histone neighborhood were identified by performing reciprocal best hits for each genome against *T. kodakarensis* using BLAST (Altschul et al. 1990), retaining those that have a similarity score of >40% and are within 20% length of one another (Rocha 2006). Note that the reciprocal best hit approach was only applied to identify putative orthologs in the neighborhood of histone genes. The histones themselves were identified using HMMER searches (see above) and subsequent analyses not restricted to reciprocal best hits of HTkA/B. To generate sequence logos, histones were aligned using MAFFT (Katoh and Standley 2013) (–localpair --maxiterate 1000) and visualized using ggseqlogo in R (Wagih 2017).

### Predicted DNA Binding and Tetramerization

Predicted DNA-binding and interaction (tetramerization) strength between dimers for Thermococcales species was computed as in Stevens et al. (2020). In brief, sequences were aligned to HMfB and substitutions were mapped onto a tetrameric model of HMfB (extracted from PDB structure 5t5k) using FoldX (Schymkowitz et al. 2005) to generate models of homotetramers with DNA. Structures were then energy-minimized using AmberTools (Maier et al. 2015) and binding affinity was calculated using an MMPBSA approach with the ff14SB forcefield (Miller et al. 2012). ΔΔG was calculated relative to HMfB. The mean value for five replicates is shown for each model.

### Species Tree

The archaeal species tree was downloaded from GTDB (https://gtdb.ecogenomic.org) on August 1, 2021.

### Expression Data

Expression data for *T. kodakarensis* were obtained from primary sources and NCBI’s Gene Expression Omnibus (GEO) (Barrett et al. 2013). Proteomics data from (Sas-Chen et al. 2020) was processed as in Hocher et al. (2021). Protein abundance at 85°C is shown. Transcript abundance data were obtained from Jäger et al. (2014) and were shown as transcripts per million.

### Bacteria-Type Singlet HF and HTkA/B Structure Prediction

The AlphaFold v2.0 (Jumper et al. 2021) collab notebook (https://colab.research.google.com/github/deepmind/alphafold/blob/main/notebooks/AlphaFold.ipynb, accessed August 21, 2018) was used to predict the structure of HTkA, HTkB, and the bacterial singlet HF protein (TK1040) as homodimers and all heterodimer combinations. The MSA method used was jackhmmer, and models were ranked by PTMscore. The top model is shown for all homodimers and heterodimers. Images shown were generated using UCSF, ChimeraX developed by the Resource for Biocomputing, Visualization and Informatics at the University of California, San Francisco, with support from National Institutes of Health (R01-GM129325) and the Office of Cyber Infrastructure and Computational Biology, National Institute of Allergy and Infectious Diseases (Pettersen et al. 2021).

### Identification of Doublet Histones in Archaea

The genomes, protein sets, and GFF files for a balanced set of archaea species were downloaded on May 21, 2021 from GenBank (https://www.ncbi.nlm.nih.gov/assembly) using taxid 2157 and processed as above (see Identification of Histones in Thermococcales Genomes). Doublet histones, including doublets from Halobacteria (Dulmage et al. 2015) and HMk in *Methanopyrus kandleri* (Fahrner et al. 2001; Slesarev et al. 1998), were identified by their position on a coding sequence-level tree of archaeal histones, and by length. This larger tree was built from 20 ML trees using Raxml-NG (Kozlov et al. 2019). Trees for the doublet histones were then built as previously described (see Classification of Thermococcales Histones into HTkA-Like and HTkB-Like Groups) and all figures plotted using iTOL (Letunic and Bork 2019). Annotation for the orthologous genes in the genomic neighborhood was generated using GeneSpy (Garcia et al. 2019). Clades with doublet histones are annotated on a tree of archaea adapted from (Borrel et al. 2020) to include Hodarchaeota (Liu et al. 2021).

### Percentage Identity of HTkA-/HTkB-Like and Bacteria-Type Histones

HTkA-like, HTkB-like and bacteria-type histones were aligned using MAFFT (Katoh and Standley 2013) (–localpair --maxiterate 1000) separately for species containing either bacteria-type doublet or bacteria-type singlet histones. For each histone type, pairwise sequence identity was calculated using seqidentity from the R package bio3d (Grant et al. 2006).

## Supplementary Material

Supplementary data are available at *Genome Biology and Evolution* online.

## Acknowledgments

This work was funded through UKRI Medical Research Council funding (MC-A658-5TY40) to T.W.

## Data Availability

All data underlying were derived from sources in the public domain: GenBank (https://www.ncbi.nlm.nih.gov/assembly), GTDB (https://gtdb.ecogenomic.org) and data from original publications cited throughout the manuscript.

## Supplementary Material

evab274_Supplementary_Data
